# Outcomes of Acute Achilles Tendon Repair with Platelet-Rich Plasma Augmentation: A Comprehensive Review of Comparative Studies

**DOI:** 10.3390/jcm15114148

**Published:** 2026-05-27

**Authors:** Michele Mercurio, Giorgia Lucia Benedetto, Elvira Immacolata Parrotta, Giovanni Cuda, Lorena Paola, Erminia Cofano, Umile Giuseppe Longo, Simone Cerciello, Giorgio Gasparini

**Affiliations:** 1 Department of Orthopaedic and Trauma Surgery, “Magna Graecia” University, “Renato Dulbecco” University Hospital, 88100 Catanzaro, Italy; michele.mercurio@unicz.it (M.M.); gasparini@unicz.it (G.G.); 2 Research Center on Musculoskeletal Health, MusculoSkeletalHealth@UMG, “Magna Graecia” University, 88100 Catanzaro, Italy; lorena.paola@studenti.unicz.it (L.P.); erminia.cofano@studenti.unicz.it (E.C.); 3 Department of Medical and Surgical Sciences, “Magna Graecia” University, 88100 Catanzaro, Italy; parrotta@unicz.it; 4 Department of Experimental and Clinical Medicine, “Magna Graecia” University, 88100 Catanzaro, Italy; cuda@unicz.it; 5 Fondazione Policlinico Universitario Campus Bio-Medico, 00128 Roma, Italy; g.longo@policlinicocampus.it; 6 Research Unit of Orthopaedic and Trauma Surgery, Department of Medicine and Surgery, Università Campus Bio-Medico di Roma, Via Alvaro del Portillo, 21, 00128 Roma, Italy; 7 Saint Camillus International University of Health and Medical Sciences, 00131 Roma, Italy; simone.cerciello@me.com

**Keywords:** Achilles tendon rupture, biological augmentation, functional outcomes, platelet-rich plasma, surgical repair, tendon healing

## Abstract

**Background/Objectives**: Platelet-rich plasma (PRP) has been proposed as a biological augmentation strategy in the surgical repair of acute Achilles tendon rupture (ATR). However, clinical evidence remains heterogeneous, and its impact on structural and functional recovery is unclear. This review synthesizes comparative clinical evidence on PRP augmentation in surgical acute ATR repair. **Methods**: A comprehensive review of comparative clinical studies was conducted using PubMed, MEDLINE, Scopus, and Cochrane Central databases. Seven studies, including three randomized controlled trials, met the inclusion criteria, totaling 207 patients. Methodological quality was assessed using the Modified Newcastle-Ottawa Scale and Cochrane Risk of Bias tool. **Results**: Several studies reported early functional improvements with PRP. Zou et al. found better range of motion at 24 months (*p* < 0.001). Sánchez et al. reported faster recovery of range of motion (7 vs. 11 weeks) and earlier return to running (11 vs. 18 weeks). Hung et al. observed improved range of motion at 6 months, without differences at 24 months (ATRS 92.9 vs. 92.7). Conversely, randomized trials by Yasui, Schepull, and De Carli showed no significant long-term differences in functional outcomes. **Conclusions**: PRP may enhance early recovery after surgical acute ATR repair, but consistent long-term superiority has not been demonstrated. Heterogeneity in PRP preparation and administration likely contributes to conflicting findings. Further standardized trials are required to define its clinical role.

## 1. Introduction

Acute Achilles tendon rupture (ATR) is one of the most common tendon injuries in active adults, with increasing incidence in recreational and middle-aged athletes [1]. Although surgical repair restores tendon continuity, functional recovery remains variable, and deficits in calf strength and return-to-sport rates are frequently reported [2]. Tendon healing is a slow and biologically complex process that rarely restores the native structural and mechanical properties of healthy tissue. The Achilles tendon is characterized by limited intrinsic vascularity and a highly organized extracellular matrix composed predominantly of type I collagen [3]. Following rupture and repair, healing proceeds through inflammatory, proliferative, and remodeling phases [4]. During the early inflammatory phase, cytokine release and cellular infiltration initiate matrix deposition. The proliferative phase is characterized by increased fibroblast and tenocyte activity, with predominant synthesis of type III collagen and neoangiogenesis. In the subsequent remodeling phase, gradual replacement of type III with type I collagen and fibrillar realignment occurs; however, complete structural restoration is rarely achieved, and scar-mediated healing predominates [5].

Given these biological limitations, strategies aimed at enhancing early cellular activity and extracellular matrix synthesis have attracted considerable interest. Platelet-rich plasma (PRP) is an autologous blood-derived product containing supraphysiological concentrations of platelets and associated growth factors, including platelet-derived growth factor (PDGF), transforming growth factor-β (TGF-β), vascular endothelial growth factor (VEGF), insulin-like growth factor-1 (IGF-1), and epidermal growth factor (EGF) [6]. These mediators are known to modulate tenocyte proliferation, angiogenesis, and collagen synthesis, suggesting a potential role in augmenting tendon repair [7,8].

Despite a strong biological rationale, clinical studies investigating PRP augmentation in acute ATR have reported inconsistent results. Biological augmentation refers to the use of biologically active substances aimed at enhancing tissue healing and regeneration. This review aims to synthesize the current clinical evidence regarding PRP augmentation in the surgical repair of acute Achilles tendon rupture reported in comparative studies.

## 2. Materials and Methods

### 2.1. Search Strategy and Study Selection

**Figure 1:**
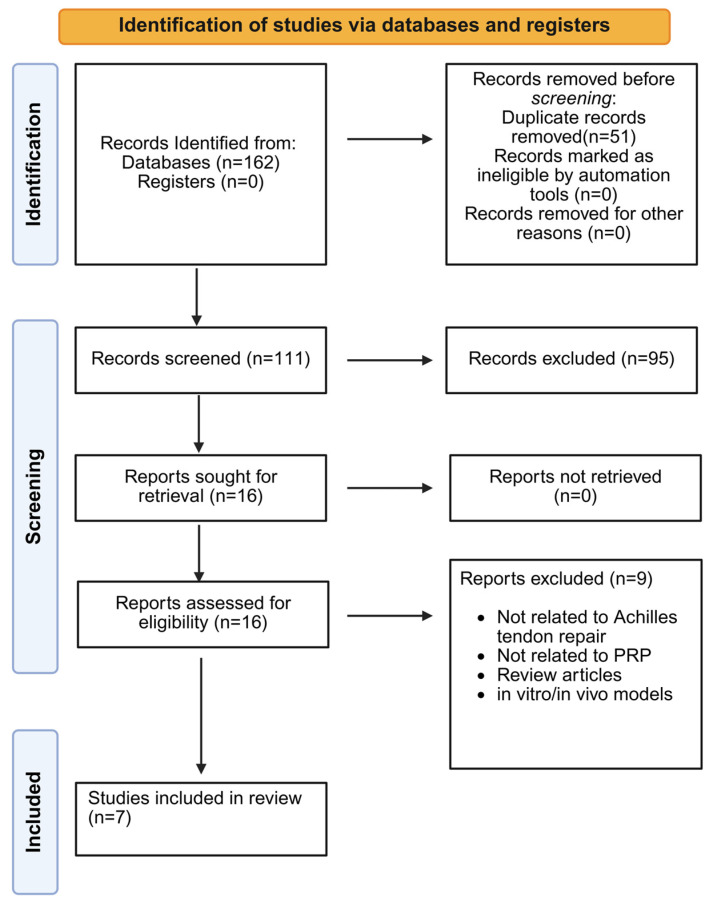
Study selection flow diagram. A total of 162 records were identified. After screening, 16 full-text articles were assessed for eligibility, and 7 studies were included in the final qualitative synthesis.

This study was conducted as a structured narrative review based on a systematic literature search. While a comprehensive and reproducible search strategy was implemented, this work was not designed as a formal systematic review. The methodology was informed by Preferred Reporting Items for Systematic Review and Meta-Analysis (PRISMA), which were applicable, although a formal systematic review design was not adopted. To improve transparency in study identification and selection, selected PRISMA principles were applied where appropriate, particularly in reporting the search process and the study selection flow. However, full adherence to PRISMA reporting standards was not intended [9]. The PubMed, MEDLINE, Scopus, and Cochrane Central databases were searched in December 2025 with no lower date limit. The search strategy included combinations of keywords and Medical Subject Headings (MeSH) terms. Boolean operators (“AND”, “OR”) were used to combine search terms, and duplicates were removed before screening. The terms “Achilles tendon rupture,” “Achilles tendon repair,” “platelet-rich plasma,” “PRP,” “platelet-rich fibrin,” and “PRGF” were used in different combinations to retrieve relevant articles. The articles were selected based on the following PICO model [10]: (P) patients who underwent acute ATR; (I) PRP augmentation; (C) patients who did not undergo additional PRP augmentation; and (O) patients assessed for functional or structural outcomes. Two authors (L.P. and G.L.B) independently conducted all the searches and screened the titles and abstracts to identify articles for inclusion. If a study could not be excluded based on the title and abstract, both reviewers reviewed the full text to reach a consensus on the inclusion or exclusion of the study by contacting a third author (M.M.) in case of major discrepancies [11]. The reference list of each included article and the available gray literature at our institution were screened for the inclusion of potential additional articles. No protocol registration was performed. Due to heterogeneity in study design, PRP formulations, and outcome measures, a meta-analysis was not feasible. The study selection process is illustrated in the study selection flow diagram (Figure 1).

### 2.2. Eligibility Criteria

Studies were included if they met the following criteria: (1) Clinical investigations involving surgical repair of acute ATR. (2) PRP used as biological augmentation. (3) Presence of a comparative control group. (4) Reporting of functional, biomechanical, or imaging outcomes. (5) reported >10 surgically treated cases. (6) written in English. The exclusion criteria were as follows: (1) studies involving isolated PRP augmentation, and (2) revision treatment of ATR. Other reviews, case reports, in vitro, cadaveric or biomechanical studies, technical notes, editorials, letters to the editor, and expert opinions were excluded from the analysis but considered for the Discussion section.

### 2.3. Study Characteristics and Data Synthesis

Two authors (L.P. and G.L.B.) independently performed a comprehensive data extraction from all included studies. The study identification and selection process incorporated selected PRISMA-based reporting elements to improve transparency. The following information was collected: first author, journal name, year of publication, study design, patient demographics, type of surgery, and follow-up period. Outcome measures included isokinetic strength testing, range of motion (ROM), return-to-sport rates, patient-reported outcome measures (ATRS, VISA-A, FAOS, SF-36), gait analysis, biomechanical elasticity assessment, and imaging evaluation via ultrasound or MRI (Table 1).

The methodological quality of the studies was independently assessed by three authors (L.P., G.L.B., and M.M.). For observational studies, the modified Newcastle-Ottawa Quality Assessment Scale (NOS) was used (Table 2). Randomized controlled trials were evaluated using the Cochrane Risk of Bias 2.0 (RoB 2.0) tool (Table 3). Discrepancies in scoring were resolved through discussion among the reviewers to reach consensus. Substantial interobserver agreement (Cohen’s kappa coefficients ranging between 0.57 and 0.72) was reported.

Although no randomized trial demonstrated critical methodological flaws, some studies raised concerns regarding allocation concealment, blinding procedures, selective outcome reporting, and limited sample size. Accordingly, Yasui et al. [15] was considered at overall low risk of bias, whereas Schepull et al. and Zou et al. [17,18] were judged as presenting some concerns according to the RoB 2.0 framework. Therefore, the findings should be interpreted with appropriate caution. Due to differences in study designs, patient populations, and outcome measures, pooled statistical analysis was not feasible. Therefore, the findings were summarized narratively, focusing on recurring clinical patterns and trends.

### 2.4. PRP Characteristics and Qualitative Synthesis

Considerable variability was observed in PRP preparation and administration. Some studies employed liquid PRP injected intraoperatively at the repair site, whereas others used platelet-rich fibrin (PRF/PRGF) matrices as biological scaffolds. Platelet concentration ranged from approximately six- to ten-fold baseline when reported, but detailed biological characterization was inconsistently provided. Leukocyte content and activation methods were frequently unspecified. Timing of administration varied from immediate intraoperative delivery to additional postoperative injections at 14 days or 3 weeks (Table 4). Given the heterogeneity of PRP preparation methods, platelet concentration, leukocyte content, timing of administration, and outcome measures, pooled statistical analysis was not feasible. Therefore, findings were synthesized narratively, with an emphasis on identifying recurring clinical patterns and interpreting results in light of tendon-healing biology. A structured qualitative synthesis was performed, focusing on early functional outcomes, long-term outcomes, and imaging findings.

## 3. Results

### 3.1. Study Overview

Seven studies met the inclusion criteria, with a total of 207 patients analyzed. Study designs comprised three randomized controlled trials (Zou et al., Schepull et al., Yasui et al.) [15,17,18], one prospective comparative study and a prospective study (De Carli et al., Hung et al.) [13,14], one retrospective biomechanical study (Alviti et al.) [12], and one case–control study involving athletes (Sánchez et al.) [16]. Across studies, substantial heterogeneity was observed in PRP preparation methods, platelet concentration, leukocyte content, activation protocols, and timing of administration. PRP was delivered intraoperatively in most studies, whereas in others it was administered postoperatively (14 days or 3 weeks after surgery). Due to substantial heterogeneity across studies, a quantitative meta-analysis was not performed. Specifically, heterogeneity was observed across multiple outcome domains: patient-reported outcome measures (ATRS, VISA-A, FAOS, SF-36) were inconsistently reported at different time points; range of motion assessments varied in methodology and units; return-to-sport outcomes were defined heterogeneously; imaging outcomes lacked standardized quantitative parameters; and complication rates were infrequently reported with low event rates. Therefore, a structured qualitative synthesis was considered more appropriate.

### 3.2. Early Functional Outcomes

Several studies reported improvements in early postoperative parameters following PRP augmentation. Zou et al. [18] demonstrated superior isokinetic muscle strength at 3 months in the PRP group. Sánchez et al. [16] observed earlier recovery of ROM and faster return to gentle running and training activities among athletes treated with platelet-rich fibrin matrices. Hung et al. [14] reported improved ankle ROM and calf circumference ratio at 6 months in the PRP group. Additionally, Alviti et al. [12], through gait analysis at 6 months, found that patients treated with platelet-rich fibrin demonstrated biomechanical parameters closer to healthy controls compared to patients treated with conventional repair alone. Conversely, other randomized trials did not confirm these early benefits. De Carli et al. [13] found no differences in pain, functional scores, isokinetic performance, or imaging findings at early follow-ups. Schepull et al. [17] reported no significant improvement in early biomechanical properties such as elasticity modulus, and Yasui et al. [15] observed no significant differences in time to achieve unilateral heel raises (Table 5).

### 3.3. Mid- to Long-Term Functional Outcomes and Return to Sport

Long-term follow-up findings were largely comparable between PRP-augmented repairs and conventional surgery. Schepull et al. [17] demonstrated no significant difference in heel raise index or elasticity modulus at one year. De Carli et al. [13] reported no differences in clinical or functional outcomes up to 6 months. Hung et al. [14] similarly found no significant between-group differences in ATRSs, return-to-sport rates, calf circumference ratio, or ROM at two years. Yasui et al. [15] in a double-blind randomized controlled trial, found no superiority of PRP over saline injection at any time point up to two years postoperatively. Although Zou et al. [18] reported improvements in ankle ROM, at 24 months, the PRP group demonstrated significantly lower deficits compared to the control group in both plantar flexion (−1.1 ± 0.3° vs. −2.0 ± 0.4°) and dorsiflexion (−1.0 ± 0.4° vs. −1.9 ± 0.4°) (*p* < 0.001). Sánchez et al. [16] reported faster functional recovery in athletes treated with PRGF, including earlier recovery of range of motion (7 ± 2 vs. 11 ± 3 weeks, *p* = 0.025) and shorter time to resume running (11 ± 1 vs. 18 ± 3 weeks, *p* = 0.042) and training activities (14 ± 0.8 vs. 21 ± 3 weeks, *p* = 0.004). However, these findings were not consistently supported across other randomized studies (Table 5).

### 3.4. Biomechanical and Imaging Findings

Biomechanical and imaging outcomes provided additional insight but remained inconsistent. Schepull et al. [17] using roentgen stereophotogrammetric analysis and computed tomography, found no significant differences in tendon elasticity modulus between PRP and control groups, despite notable interindividual variability. Alviti et al. [12], however, reported improved gait efficiency parameters in patients treated with platelet-rich fibrin matrices. De Carli et al. [13] observed reduced gadolinium enhancement on MRI at 6 months in the PRP group, although this finding was not associated with clinically relevant differences, with postoperative pain scores remaining comparable between groups (VAS: 0.3 vs. 0.6). Sánchez et al. [16] reported a smaller increase in tendon cross-sectional area in patients treated with PRGF (298% ± 90% vs. 499% ± 91%), suggesting a potential modulation of tendon remodeling. However, Yasui et al. [15] did not identify any significant MRI differences between groups (ROM: 1.8 ± 0.5 vs. 1.5 ± 0.5) (Table 6).

## 4. Discussion

### 4.1. Overview of the Evidence

This review examined the available comparative clinical studies evaluating PRP augmentation in the surgical repair of acute ATR. The overall evidence reveals a heterogeneous pattern. Some investigations report early improvements in postoperative functional parameters, whereas the majority of randomized trials do not demonstrate consistent long-term superiority over repair alone [12,13,14,15,16,17,18]. This variability in the results reported could also be influenced by preparation protocols and timing of administration. The absence of quantitative synthesis reflects the substantial heterogeneity across included studies. Differences in outcome definitions, measurement methods, and follow-up timing across domains such as patient-reported outcomes, range of motion, return to sport, and imaging prevented meaningful data pooling.

### 4.2. Early Recovery and Biological Rationale

The biological rationale for PRP augmentation is grounded in the physiology of tendon healing [19,20]. During the inflammatory and early proliferative phases, growth factors such as platelet-derived growth factor (PDGF), transforming growth factor-β (TGF-β), vascular endothelial growth factor (VEGF), and insulin-like growth factor (IGF-1) contribute to cell recruitment, angiogenesis, and extracellular matrix synthesis [21]. Some studies reported early improvements; however, these findings were inconsistent, often derived from small sample sizes, and should be interpreted cautiously given the potential risk of type I error These findings are biologically plausible and may reflect transient enhancement of cellular activity and early collagen deposition. However, such effects were not consistently observed across all trials and were often limited to short-term follow-ups.

### 4.3. Lack of Durable Clinical Benefit and Clinical Relevance

One of the most consistent findings across the literature is the absence of durable long-term superiority in PRP-augmented repairs [22]. At mid- and long-term follow-up, patient-reported scores, return-to-sport rates, and objective functional assessments are generally comparable between PRP and control groups. Comparative evidence from both randomized and non-randomized studies generally supports the absence of consistent long-term superiority of PRP augmentation despite occasional early or transient benefits reported in some studies [12,13,14,15,16,17,18]. This observation highlights the central role of mechanical loading [23] and rehabilitation in tendon remodeling. The maturation phase of tendon healing is driven by collagen alignment, cross-linking, and mechano-transduction pathways, processes that are strongly influenced by progressive loading rather than solely by biochemical stimulation [24,25]. While PRP may enhance early matrix synthesis, it does not directly regulate fibrillar organization or long-term structural adaptation. An additional consideration is the clinical relevance of the reported between-group differences. Although some studies reported statistically significant improvements in outcomes such as ankle range of motion or Achilles Tendon Total Rupture Score (ATRS), these differences were often small and should be interpreted in light of minimal clinically important differences (MCID). For ATRS, reported differences in several randomized studies may be too small to represent meaningful patient benefit despite statistical significance. Similarly, modest gains in range of motion, although potentially indicative of accelerated early recovery, may have uncertain functional relevance when considered in long-term outcomes. Given the lack of validated MCID specifically established for several outcomes in acute Achilles tendon rupture repair, the clinical importance of these observed differences remains uncertain and warrants cautious interpretation. For example, Zou et al. [18] and Hung et al. [14] reported ATRS differences of 2–3 points, which remain below commonly proposed thresholds for clinical relevance, such as the 10-point threshold proposed in the original ATRS validation study by Nilsson-Helander et al. [26]. Overall, re-tear rates across studies were consistently low and comparable between PRP and control groups, with no clear advantage of PRP augmentation. Some imaging-based findings suggested potential modulation of tendon remodeling in PRP-treated patients, including differences in gadolinium enhancement or tendon cross-sectional area. However, these structural observations were not consistently associated with superior mechanical performance or patient-reported outcomes and should therefore be interpreted cautiously.

### 4.4. Biological Heterogeneity of PRP Preparations

A major limitation of the current evidence base is the heterogeneity in PRP preparation and application. Platelet-rich plasma (PRP), platelet-rich fibrin (PRF), and plasma rich in growth factors (PRGF) represent biologically distinct formulations. PRP is typically liquid, PRF forms a fibrin matrix scaffold, while PRGF is characterized by reduced leukocyte content. These differences may influence healing dynamics. PRP formulations varied substantially across studies. Differences in leukocyte content (leukocyte-rich vs. leukocyte-poor), timing of administration (intraoperative vs. delayed), and formulation (PRP vs. PRF vs. PRGF) may contribute to the heterogeneity of clinical outcomes. PRF-based approaches appeared to provide a more stable fibrin scaffold, while liquid PRP showed more variable results. Similarly, leukocyte-rich preparations may induce a stronger inflammatory response, potentially influencing early healing phases. Variability in platelet concentration, leukocyte content, activation methods, use of fibrin matrices, injected volume, and timing of administration suggests that the term “PRP” encompasses biologically distinct products. Few studies provided a detailed characterization of platelet counts or growth factor concentrations, and leukocyte content was inconsistently reported. Given that leukocyte-rich and leukocyte-poor preparations may exert different inflammatory and anabolic effects, this variability is likely to influence clinical outcomes [19,27]. Moreover, the optimal biological dose and timing relative to the healing cascade remain undefined. Beyond representing methodological variability, these biological differences may partly explain the inconsistent clinical outcomes reported across studies. For example, fibrin-based products such as PRF or PRGF may provide a scaffold effect supporting cell migration and early matrix organization, potentially contributing to some of the early functional improvements observed in studies using these formulations. In contrast, liquid PRP preparations may act predominantly through transient growth factor delivery, which could explain more variable or short-lived effects. Likewise, leukocyte-rich preparations may enhance early inflammatory signaling but potentially at the cost of excessive catabolic responses, whereas leukocyte-poor formulations may favor a more anabolic environment. These differences suggest that variability in clinical outcomes may reflect product-specific biology rather than inconsistent efficacy of “PRP” as a single intervention. Early intraoperative delivery may target the inflammatory phase, whereas delayed postoperative injection may interact differently with proliferative or remodeling processes. Without protocol standardization, drawing definitive conclusions regarding efficacy remains challenging.

### 4.5. Clinical Implications and Future Directions

While some studies report early functional benefits, consistent long-term superiority has not been demonstrated in high-quality randomized trials. The absence of consistent long-term benefits should not be equated with definitive ineffectiveness. Instead, it highlights the need for biologically driven trial designs, including standardized PRP characterization, controlled dosing strategies, and integration with optimized rehabilitation protocols. Future research should also investigate whether specific patient subgroups, such as high-demand athletes or individuals with impaired healing capacity, might derive greater benefit from PRP augmentation. Taken together, the available evidence supports a conceptual framework in which platelet-derived products may primarily influence the early inflammatory and proliferative phases of tendon healing, potentially improving early matrix formation and functional recovery in selected contexts, whereas long-term outcomes appear predominantly governed by mechanobiological remodeling and rehabilitation. This translational perspective may help reconcile the discrepancy between occasional early benefits and the absence of consistent durable superiority across clinical trials. 

### 4.6. Limitations

Several limitations of the present study should be acknowledged. First, only articles published in English were included, which may have introduced a potential publication bias. Although four major databases were systematically searched, the possibility remains that relevant studies may have been missed. Second, the included studies showed variability in follow-up duration, despite all reporting a minimum follow-up of 6 months. Functional outcomes and complication rates may be influenced by the length of follow-up, and different results might emerge with longer or more standardized observation periods. Third, substantial heterogeneity was observed among the included studies in terms of sample size, patient characteristics, and lesion type, with a higher rate of midportion tendon injuries and physically active male patients. This variability may limit the generalizability of the findings and should be considered when interpreting the results. In addition, heterogeneity in surgical techniques and post-operative rehabilitation protocols was noted, and the lack of sufficiently detailed or comparable data prevented a subgroup analysis based on the type of surgical intervention. Differences in patient-specific factors and treatment strategies may influence outcomes and should be carefully considered in clinical decision-making. Furthermore, variability in PRP preparation methods, composition, and timing of administration represents a confounding factor when interpreting clinical outcomes. Finally, although radiological and functional outcomes are commonly reported, there is a lack of comparative studies specifically addressing histological outcomes, which may provide further insight into tendon healing processes. Overall, the current literature does not provide definitive evidence of a sustained functional advantage, but neither does it conclusively exclude a potential role of PRP when applied under optimized conditions.

## 5. Conclusions

A biological rationale supports PRP augmentation in acute Achilles tendon repair and may provide early or context-dependent benefits. However, current comparative evidence does not demonstrate consistent long-term clinical superiority over standard repair. Given the substantial heterogeneity in PRP formulations and study designs, routine use cannot be supported based on the available data. Future well-designed trials with standardized PRP protocols and clinically meaningful outcome measures are required to better define its role.

## Figures and Tables

**Table 1:** Characteristics of included studies.

Author and Year	Journal	Scientific Level	Study Design and Years	N. Patients	PRP Group	Control Group	Age Mean ± SD or Range	Follow-Up	Type of Surgery/PRP Method	Outcome Measures
Alviti et al., 2017 [12]	J Foot Ankle Surg,	IV	Retrospective, 2010–2014	20	11	9	PRP group: 32.5 ± 3.4; Control group: 34.5 ± 3	6	Open repair ± PRF	Gait analysis, ankle biomechanics
De Carli et al., 2016 [13]	Knee Surg Sports Traumatol Arthrosc	IV	Prospective comparative, 2011	30	15	15	32 ± 0 range: 25–37 years	24	Mini-open + percutaneous ± PRP	VAS, FAOS, VISA-A, US, MRI, Isokinetic/jumping tests
Hung et al., 2022 [14]	J Clin Med	II	Prospective, 2014–2018	62	32	30	PRP group 37.5± 8.7 (range 22–50 years); Control group 39.6 ± 8.5 (range 20–53 years)	24	Endoscopy-assisted percutaneous ± PRP	ATRS, ROM, calf circumference, return-to-sport
Yasui et al., 2025 [15]	J Exp Orthop	I	Double-blind RCT, 2018–2021	14 analyzed (17 enrolled; 3 excluded)	7	7	Range: 30–59 years	24	Side-locking loop ± PRP	Heel raise tests, jogging initiation, MRI
Sánchez et al., 2007 [16]	Am J Sports Med	III	Case–control, 1997–2004	12	6	6	PRP group: 36.2 ± 6.2; Control group: 32.1 ± 6	12	Open repair + venipuncture PRGF	ROM, return-to-sport, US, growth factor analysis
Schepull et al., 2011 [17]	Am J Sports Med	II	RCT, 2007–2008	30	16	14	PRP group: 39.8 ± 6.2; control group: 39.4 ± 8.3 (range 18–60 years)	12	Open repair + venipuncture PRP	Elasticity modulus, heel raise index, ATRS
Zou et al., 2016 [18]	BioMed Res Int	II	RCT, 2013–2014	36	16	20	PRP group: 30.2 ± 5.8; control group: 28.9 ± 5.7 (range 18–45 years)	24	Open repair + venipuncture PRP	Isokinetic strength, ROM, Leppilahti, SF-36

Main characteristics of the included clinical studies evaluating PRP augmentation in the surgical repair of acute Achilles tendon rupture. Study design, sample size, surgical technique, PRP formulation, timing of application, follow-up, and outcomes are summarized. Abbreviation: ATRS, Achilles Tendon Total Rupture Score; FAOS, Foot and Ankle Outcome Score; MRI, magnetic resonance imaging; PRP, Platelet-rich plasma; RCT, randomized clinical trials; ROM, range of motion; SF-36, Short Form (36) Survey; US, Ultrasound; VAS, Visual Analogic Scale; VISA-A, Victorian Institute of Sports Assessment-Achilles.

**Table 2:** Newcastle-Ottawa Scale (NOS).

Study	Selection (Max 4)	Comparability (Max 2)	Outcome (Max 3)	Total Score (/9)	Risk of Bias
Alviti et al., 2017 [12]	3	1	2	6	Moderate
De Carli et al., 2016 [13]	3	1	2	6	Moderate
Hung et al., 2022 [14]	3	2	3	8	Low
Sánchez et al., 2007 [16]	2	1	2	5	Moderate

The Newcastle-Ottawa Scale assesses observational studies on three domains: selection of study groups, comparability, and outcome assessment. Scores range from 0 to 9, with higher values indicating higher methodological quality. Studies scoring 7–9 are considered at low risk of bias, 5–6 at moderate risk, and ≤4 at high risk.

**Table 3:** Cochrane risk-of-bias tool for randomized trials (ROB 2.0).

Study	Randomization Process	Deviations from Intended Interventions	Missing Outcome Data	Measurement of Outcome	Selection of Reported Result	Overall Risk of Bias
Yasui et al., 2025 [15]	Low	Low	Low	Low	Low	Low
Schepull et al., 2011 [17]	Some concerns	Low	Low	Low	Some concerns	Some concerns
Zou et al., 2016 [18]	Some concerns	Low	Low	Low	Some concerns	Some concerns

The Cochrane Risk of Bias 2.0 tool evaluates randomized controlled trials across five domains: the randomization process, deviations from intended interventions, missing outcome data, measurement of the outcome, and selection of the reported result. Each domain is rated as low risk of bias, some concerns, or high risk of bias. An overall risk of bias is then determined based on these ratings.

**Table 4:** Characteristics of PRP protocols in different studies.

Author (Year)	PRP Type	Activation	Timing	Volume
Alviti et al., 2017 [12]	PRF matrix	Ca-gluconate + batroxobin	Intraoperative	NR
De Carli et al., 2016 [13]	Liquid PRP + gel	Thrombin + Ca-gluconate	Intraop + 14 days	4 mL + 2 mL
Hung et al., 2022 [14]	Liquid PRP	None	Intraop + 2 weeks	4 mL
Yasui et al., 2025 [15]	Liquid PRP	None	3 weeks post-op	NR
Sánchez et al., 2007 [16]	PRGF	Calcium chloride	Intraoperative	4 mL + 4 mL
Schepull et al., 2011 [17]	Liquid PRP	None	Intraoperative	21 mL
Zou et al., 2016 [18]	Liquid PRP	None	Intraoperative	3–4 mL

Summary of PRP preparation in the included studies. Abbreviation: PRP, platelet-rich plasma; PRF, platelet-rich fibrin; PRGF, plasma rich in growth factors; NR, not reported; Ca-gluconate, calcium gluconate; post-op, postoperative.

**Table 5:** Key quantitative functional outcomes following PRP augmentation.

Author (Year)	Key Outcomes	Selected Quantitative Findings PRP vs. Control
Alviti et al., 2017 [12]	Gait	Favorable vs. no-PRF
De Carli et al., 2016 [13]	FAOS, VAS	No difference (FAOS: 92.7 vs. 93.4; VAS: 0.3 vs. 0.6)
Hung et al., 2022 [14]	ATRS, ROM	No long-term difference (ATRS: 92.9 vs. 92.7)
Sánchez et al., 2007 [16]	Tendon structure (CSA)	Favorable (structural) (CSA: 298% ± 90% vs. 499% ± 91%)
Schepull et al., 2011 [17]	Heel raise, ATRS	No difference (ATRS: 78 vs. 89)
Yasui et al., 2025 [15]	ROM, ATRS	No difference (ROM: 1.8 ± 0.5 vs. 1.5 ± 0.5; ATRS: 99 vs. 99)
Zou et al., 2016 [18]	ROM, ATRS	Favorable (early and long-term) (ATRS: 88 vs. 85; ROM (DF): −1.0 ± 0.4° vs. −1.9 ± 0.4°)

Summary of the main functional outcomes reported in studies evaluating platelet-rich plasma (PRP) or platelet-rich fibrin (PRF) augmentation in surgical Achilles tendon repair. Only quantitative data available from the extracted dataset are reported. Results include selected functional scores and objective measures where available. Abbreviation: ATRS, acute Achilles tendon rupture score; CSA, cross-sectional area; DF, dorsiflexion. FAOS, Foot and Ankle Outcome Score; PRP, platelet-rich plasma; PRF, platelet-rich fibrin; ROM, range of motion; VAS, Visual Analogic Scale.

**Table 6:** Biomechanical and Imaging Outcomes after PRP Augmentation.

Author (Year)	Biomechanical/Gait Analysis	Imaging (US/MRI)	PRP vs. Control Results	Selected Quantitative Findings
De Carli et al., 2016 [13]	NR	MRI (gadolinium), US	Reduced enhancement in PRP	VAS: 0.3 vs. 0.6
Sánchez et al., 2007 [16]	NR	US: tendon CSA	Smaller CSA in PRGF group	CSA: 298% ± 90% vs. 499% ± 91%
Yasui et al., 2025 [15]	NR	MRI	No significant difference	ROM: 1.8 ± 0.5 vs. 1.5 ± 0.5

Imaging-based assessment of tendon healing after PRP augmentation. Ultrasound and magnetic resonance imaging findings are summarized, including tendon cross-sectional area, signal intensity, and contrast enhancement patterns. Structural differences were occasionally reported; however, these findings did not consistently correlate with improved functional outcomes. Abbreviation: PRP, platelet-rich plasma; US, ultrasound; MRI, magnetic resonance imaging; CSA, cross-sectional area; NR, not reported; VAS, Visual Analogic Scale.

## Data Availability

No new data were created or analyzed in this study. Data sharing is not applicable to this article.

## Abbreviations

The following abbreviations are used in this manuscript:

ATR	Acute Achilles tendon rupture
ATRS	Achilles Tendon Total Rupture Score
CSA	Cross-sectional area
CT	Computed tomography
EGF	Epidermal growth factor
FAOS	Foot and Ankle Outcome Score
IGF-1	Insulin-like growth factor 1
MRI	Magnetic resonance imaging
NR	Not reported
PDGF	Platelet-derived growth factor
post-op	Post operative
PRF	Platelet-rich fibrin
PRGF	Plasma rich in growth factors
PRP	Platelet-rich plasma
ROM	Range of motion
SF-36	Short Form (36) Health Survey
TGF-β	Transforming growth factor beta
US	Ultrasound
VAS	Visual Analogic Scale
VEGF	Vascular endothelial growth factor
VISA-A	Victorian Institute of Sports Assessment-Achilles

## References

[1] Delmastro E., Colace S., Longo U.G., D’Hooghe P., Marangon A., Galasso O., Gasparini G., Mercurio M. (2025). Revision Surgery for Achilles Tendon Rupture: A Comprehensive Review of Treatment Options, Outcomes, and Complications and the Role of Artificial Intelligence. Medicina.

[2] Cofano E., Colace S., Piro F., Longo U.G., D’Hooghe P., Kennedy J.G., Marangon A., Gasparini G., Mercurio M., SIAGASCOT (2025). Successful Functional Outcomes and Return to Sport Rate Can Be Achieved after Surgery for Acute Achilles Tendon Rupture: A Systematic Review. J. Exp. Orthop..

[3] Citro V., Clerici M., Boccaccini A.R., Della Porta G., Maffulli N., Forsyth N.R. (2023). Tendon Tissue Engineering: An Overview of Biologics to Promote Tendon Healing and Repair. J. Tissue Eng..

[4] Darrieutort-Laffite C., Blanchard F., Soslowsky L.J., Le Goff B. (2024). Biology and Physiology of Tendon Healing. Jt. Bone Spine.

[5] Schulze-Tanzil G.G., Delgado Cáceres M., Stange R., Wildemann B., Docheva D. (2022). Tendon Healing: A Concise Review on Cellular and Molecular Mechanisms with a Particular Focus on the Achilles Tendon. Bone Jt. Res..

[6] Mercurio M., Minici R., Spina G., Cofano E., Laganà D., Familiari F., Galasso O., Gasparini G. (2025). Clinical and Radiological Outcomes of Combined Arthroscopic Microfracture and Mesenchymal Stem Cell Injection Versus Isolated Microfracture for Osteochondral Lesions of the Talus: A Meta-Analysis of Comparative Studies. J. Foot Ankle Surg..

[7] Miron R.J., Gruber R., Farshidfar N., Sculean A., Zhang Y. (2024). Ten Years of Injectable Platelet-rich Fibrin. Periodontology 2000.

[8] Hohmann E. (2025). Editorial Commentary: High-Platelet-Dose Platelet-Rich Plasma May Be the Nonoperative Treatment of Choice for Knee Osteoarthritis. Arthroscopy.

[9] Moher D., Liberati A., Tetzlaff J., Altman D.G., The PRISMA Group (2009). Preferred Reporting Items for Systematic Reviews and Meta-Analyses: The PRISMA Statement. PLoS Med..

[10] Schardt C., Adams M.B., Owens T., Keitz S., Fontelo P. (2007). Utilization of the PICO Framework to Improve Searching PubMed for Clinical Questions. BMC Med. Inform. Decis. Mak..

[11] Page M.J., McKenzie J.E., Bossuyt P.M., Boutron I., Hoffmann T.C., Mulrow C.D., Shamseer L., Tetzlaff J.M., Akl E.A., Brennan S.E. (2021). The PRISMA 2020 statement: An updated guideline for reporting systematic reviews. BMJ.

[12] Alviti F., Gurzì M., Santilli V., Paoloni M., Padua R., Bernetti A., Bernardi M., Mangone M. (2017). Achilles Tendon Open Surgical Treatment With Platelet-Rich Fibrin Matrix Augmentation: Biomechanical Evaluation. J. Foot Ankle Surg..

[13] De Carli A., Lanzetti R.M., Ciompi A., Lupariello D., Vadalà A., Argento G., Ferretti A., Vulpiani M.C., Vetrano M. (2016). Can Platelet-rich Plasma Have a Role in Achilles Tendon Surgical Repair?. Knee Surg. Sports Traumatol. Arthrosc..

[14] Hung C.-Y., Lin S.-J., Yeh C.-Y., Yeh W.-L. (2022). Effect of Platelet-Rich Plasma Augmentation on Endoscopy-Assisted Percutaneous Achilles Tendon Repair. J. Clin. Med..

[15] Yasui Y., Miyamoto W., Sasahara J., Keisuke T., Kubo M., Sasaki G., Yamamoto A., Kawano H. (2025). No Significant Impact of Platelet-rich Plasma on Recovery after Achilles Tendon Surgery: A Double-blind Randomized Controlled Trial. J. Exp. Orthop..

[16] Sánchez M., Anitua E., Azofra J., Andía I., Padilla S., Mujika I. (2007). Comparison of Surgically Repaired Achilles Tendon Tears Using Platelet-Rich Fibrin Matrices. Am. J. Sports Med..

[17] Schepull T., Kvist J., Norrman H., Trinks M., Berlin G., Aspenberg P. (2011). Autologous Platelets Have No Effect on the Healing of Human Achilles Tendon Ruptures: A Randomized Single-Blind Study. Am. J. Sports Med..

[18] Zou J., Mo X., Shi Z., Li T., Xue J., Mei G., Li X. (2016). A Prospective Study of Platelet-Rich Plasma as Biological Augmentation for Acute Achilles Tendon Rupture Repair. BioMed Res. Int..

[19] Chalidis B., Givissis P., Papadopoulos P., Pitsilos C. (2023). Molecular and Biologic Effects of Platelet-Rich Plasma (PRP) in Ligament and Tendon Healing and Regeneration: A Systematic Review. Int. J. Mol. Sci..

[20] Kale P., Patel H., Jaiswal A.M. (2024). Mechanisms, Efficacy, and Clinical Applications of Platelet-Rich Plasma in Tendinopathy: A Comprehensive Review. Cureus.

[21] Ruiz-Alonso S., Lafuente-Merchan M., Ciriza J., Saenz-del-Burgo L., Pedraz J.L. (2021). Tendon Tissue Engineering: Cells, Growth Factors, Scaffolds and Production Techniques. J. Control. Release.

[22] Pallikkara Kuttyadan N., Samad S., Shahzad M., Sanaullah K., Gillani S.H.M., Mushtaq H., Butt S.B., Hayat S.K., Raif M., Ali S.M. (2025). Effectiveness of Platelet-Rich Plasma Injection for Chronic Achilles Tendinopathy: An Umbrella Systematic Review. Cureus.

[23] Kjaer M., Langberg H., Heinemeier K., Bayer M.L., Hansen M., Holm L., Doessing S., Kongsgaard M., Krogsgaard M.R., Magnusson S.P. (2009). From mechanical loading to collagen synthesis, structural changes and function in human tendon. Scand. J. Med. Sci. Sports.

[24] Sakai T., Kumagai K. (2025). Molecular Dissection of Tendon Development and Healing: Insights into Tenogenic Phenotypes and Functions. J. Biol. Chem..

[25] Lin M., Li W., Ni X., Sui Y., Li H., Chen X., Lu Y., Jiang M., Wang C. (2023). Growth Factors in the Treatment of Achilles Tendon Injury. Front. Bioeng. Biotechnol..

[26] Mercurio M., Castioni D., Porco E., Familiari F., Gasparini G., Galasso O. (2022). Periprosthetic Ankle Infection: Eradication Rate, Complications, and Limb Salvage. A Systematic Review. Foot Ankle Surg..

[27] Nilsson-Helander K., Thomee R., Silbernagel K.G., Thomee P., Faxen E., Eriksson B.I., Karlsson J. (2007). The Achilles tendon Total Rupture Score (ATRS): Development and validation. Am. J. Sports Med..

